# Retrospective Study of Kyasanur Forest Disease and Deaths among Nonhuman Primates, India, 1957–2020

**DOI:** 10.3201/eid2707.210463

**Published:** 2021-07

**Authors:** Sulagna Chakraborty, William E. Sander, Brian F. Allan, Flavia C.D. Andrade

**Affiliations:** University of Illinois Urbana–Champaign, Urbana–Champaign, Illinois, USA

**Keywords:** Kyasanur Forest disease, vector-borne infections, tickborne diseases, monkey, deaths, sentinel species, meningitis/encephalitis, viruses, primates, India

## Abstract

Kyasanur Forest disease (KFD) is a tickborne hemorrhagic disease affecting primates along the Western Ghats mountain range in India. Our retrospective study indicated that >3,314 monkey deaths attributed to KFD were reported in KFD-endemic states in India during 1957–2020. These data can help guide surveillance to protect animal and human health.

Kyasanur Forest disease (KFD) is a highly infectious tickborne disease affecting humans and monkeys. The etiologic agent of this disease is the Kyasanur Forest disease virus (KFDV), a flavivirus. Since its discovery in 1957 in Karnataka State, India, KFD has expanded to 5 states along the western coastline in India (*1*) and ≈10,000 reported cases of KFD in humans, averaging 400–500 cases annually (*2*). After an incubation period of 3–8 days, primary clinical symptoms include fever, myalgia, and gastrointestinal and bleeding problems. In a small subset of patients, a second phase of the disease can include neurologic manifestations and fever. If the disease is detected early, symptomatic supportive care can improve recovery from the disease. Case-fatality rates range from 3% to 15% (*1*,*3*). The primary vectors of KFDV are *Haemaphysalis spinigera* and *H. turturis *ticks, which are endemic to southern India and transmit the virus to monkeys and humans (*4*). Larvae and nymphs of these ticks feed on monkeys when the monkeys are ground foraging, providing routes of infection and spread. In addition, KFDV can be transmitted transovarially in these ticks (Figure 1).

**Figure 1:**
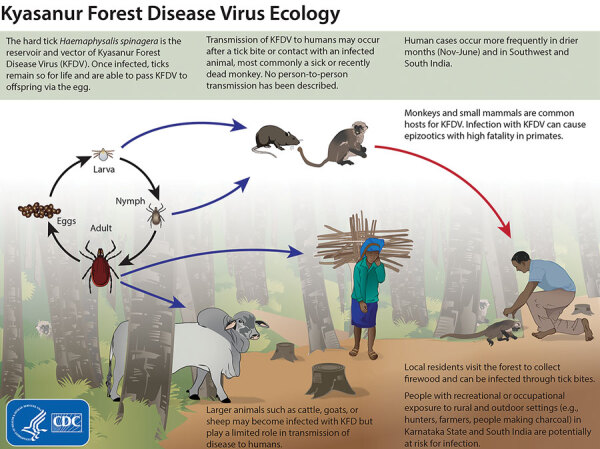
Ecology of Kyasanur Forest disease virus. Reproduced from https://www.cdc.gov/vhf/kyasanur/resources/virus-ecology.html.

*Macaca radiata* and *Semnopithecus entellus* are 2 monkey species in the KFD-endemic region frequently associated with KFD; these monkeys can succumb to the virus quickly (*3*). For monkeys, KFDV causes nonspecific and degenerative changes in abdominal organs, hemorrhage, and encephalitis. Experimentally infected monkeys have diarrhea, bradycardia, and hypotension and ultimately die (*5*). Monkey migration might expand KFDV geographic distribution, in which infected ticks are carried across state borders through connected natural areas (*1*,*3*,*4*). Although reporting of monkey deaths from KFD during the past 60 years has been unsystematic and inconsistent, the data provide valuable information. We summarize reports of monkey deaths connected with KFD in India and evaluate the utility of reporting KFD occurrence in monkeys for human disease surveillance.

## The Study

We conducted a retrospective review of scientific literature through Web of Science, PubMed Central, and Google Scholar and included data from Pro-MED Mail, newspapers, and government reports issued during 1957–2020. The search keywords included KFD, KFDV, monkey fever, Kyasanur Forest disease, and mankan kayla (a local term in Karnataka, India). We used 55 peer-reviewed journal articles, 109 Pro-MED Mail reports, 1 report by the Karnataka State government, and 1 newspaper report to generate estimates. We created a database from all information sources; our final dataset (Table 1) contains the most updated information for all years from the available data.

**Table 1:** Monkey deaths attributed to Kyasanur Forest disease in the southwestern states of India, 1957–2020*

Year	Total no. monkey deaths	No. monkey deaths, state of occurrence
1957 Jan–Sep	105	105, KN
1957–1958 Oct–Sep	92	92, KN
1958–1959	290	290, KN
1959–1960	187	187, KN
1960–1961	80	80, KN
1961–1962	114	114, KN
1962–1963	147	147, KN
1963–1964	144	144, KN
1964–1965	109	109, KN
1965–1966	191	191, KN
1967–1968	126	126, KN
1968–1969	138	138, KN
1969–1970	135	135, KN
1970–1971	88	88, KN
1971–1972	75	75, KN
1972–1973	101	101, KN
1973–1974	83	83, KN
1975–1981	No data	No data
1982–1983	>35	<35, KN
1983–1997	No data	No data
1998	Dead monkeys reported	No figure reported† for KN
1999	No data	No data
2000	Several dead monkeys reported	No figure reported for KN
2001–2002	No data	No data
2003	132	132, KN
2004	86	86, KN
2005	53	53, KN
2006	61	61, KN
2007	19	19, KN
2008	23	23, KN
2009	86	86, KN
2010	28	28, KN
2011	>35	<35, KN
2012	>64	39-64, KN; No figure reported for TN
2013	50	50, KN
2014	>131	31, KN; <100, KL
2015	60	42, KN; 18, KL
2016	72	3, MH; 69, GA
2017	>81	<51, KL; <10, GA
2018	>76	<76, KN; No figure reported for KL or MH
2019	>15	15, KN; No figure reported for KL
2020	>2	2, KN; No figure reported for KL

*GA, Goa; KL, Kerala; KN, Karnataka; MH, Maharashtra; TN, Tamil Nadu. †No figure reported indicates that monkey deaths were reported in a state but an exact number was not provided.

Information on monkey deaths caused by KFD is limited, particularly for species-specific deaths. Our review of all data sources indicates that >3,314 monkey deaths associated with KFD were reported during 1957–2020 (Table 1). However, only a subset of deaths were tested for KFDV. During this period, 760 monkeys underwent necropsy, and 334 were laboratory-confirmed to have KFDV infection (Appendix). Of the reported monkey deaths, a total of 1,676 deaths occurred in *S. entellus* monkeys and 400 deaths occurred in *M. radiata *monkeys; species were not reported for the remaining 1,238 deaths.

**Figure 2:**
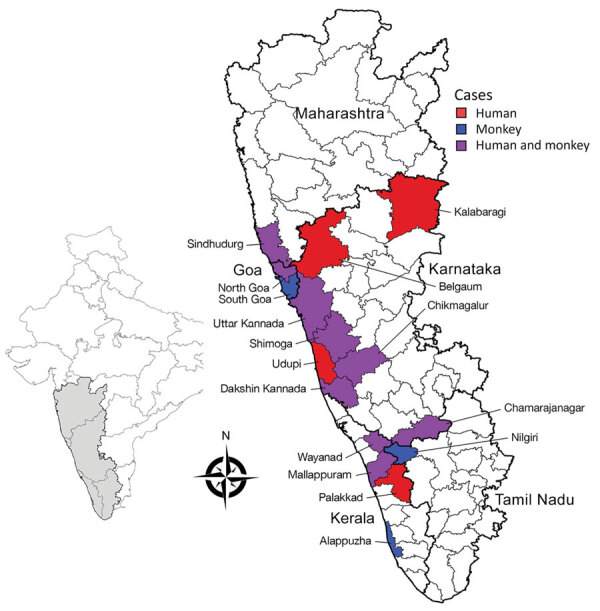
Hotspot areas for human cases and monkey deaths attributable to Kyasanur Forest disease, India,1957–2020. Inset map shows the region in context of the Indian subcontinent.

We found an early report of KFD in monkeys outside of Karnataka in Tamil Nadu state in 2012, which could be linked to an outbreak of human cases at the Bandipur Tiger Reserve in 2012. Monkeys from Karnataka might have entered Tamil Nadu carrying the virus or infected ticks. Subsequently, KFD in monkeys was reported in Kerala state in 2014 and Goa and Maharashtra states in 2016. Substantial overlap occurred between reported monkey deaths and human cases of KFD (Figure 2). We identified the drivers behind KFD transmission and geographic expansion based on the literature (Table 2).

**Table 2:** Information about potential drivers of Kyasanur Forest Disease transmission based on review of available literature

Drivers	Source of information (reference)
Large-scale deforestation for various reasons (e.g., paddy fields and plantations)	Ajesh et al., 2017 (*1*); Pattnaik, 2006 (*3*)
Human encroachment into forested areas	Pattnaik, 2006 (*3*); Murhekar et al., 2015 (*6*)
Humidity in paddy fields ideal for tick survival	Pattnaik, 2006 (*3*)
Vector ticks can survive in various kinds of biotypes	Sadanandane et al., 2018 (*4*)
Number of small mammalian animals that act as reservoirs for the virus and for the vector tick	Pattnaik, 2006 (*3*)
Movement of monkeys into new areas	Chakraborty et al., 2019 (*2*); Pattnaik, 2006 (*3*)
Cattle may act as amplifying hosts for Kyasanur Forest disease virus and help in maintenance and propagation of the tick vector (handling of cows might also be a risk factor)	Chakraborty et al., 2019 (*2*)

Higher mortality rates occurred among *S. entellus* monkeys (81% of 1,159 deaths) than among *M. radiata* monkeys during 1957–1964 (*7*). Most monkey deaths were reported in evergreen and semievergreen forests in the Western Ghats (*8*). We found no other information associating the frequency of monkey deaths to habitat.

The abundance of these primate species in the area of interest is difficult to determine because of limited studies with inconsistent sampling methods. In Karnataka, higher encounter rates with *M. radiata* monkeys were reported in wet evergreen forests and human-inhabited areas (*9*). *M. radiata* monkeys were encountered mainly in the Western Ghats and the Southern Plateau, whereas *S. entellus* monkeys were abundant in the Western Ghats and Northern Plains. Based on a 2001 Environmenta Information System bulletin (*10*), the national population of *M. radiata* monkeys in India was ≈150,000 and that of *S. entellus* monkeys was ≈300,000. Both species have suffered population decline because of habitat loss, translocation, and hunting, and minimal efforts have been undertaken to conserve these species (*9*,*11*).

## Conclusions

Our study highlights the need for consistent surveillance of monkey deaths. Monkey deaths caused by KFDV are harbingers of human cases (*1*,*3*,*4*), making these animals potential sentinels for KFD (*6*). Therefore, determining these primate species’ relative susceptibility to KFDV to evaluate the potential to use monkey deaths for surveillance is essential. In laboratory experiments, higher mortality rates have been reported in *S. entellus* than *M. radiata* monkeys (*12*). Patil et al. (*13*) experimentally infected *M. radiata* monkeys with KFDV and found that only 20% of these primates had onset of severe clinical signs, but all exhibited viral shedding and a humoral immune response. Thus, other factors might contribute to KFD mortality rates under natural conditions, and *M. radiata* monkeys might be less susceptible to KFD than previously thought. KFDV infection can often be subclinical in nature, explaining why fewer deaths have been observed for *M. radiata* than *S. entellus* monkeys. By shedding the virus through body secretions, *M. radiata* monkeys might aid in expanding KFDV into new areas. This phenomenon underscores the need for conducting serum or fecal surveillance of primates to determine KFDV epidemiology and transmission.

Most human cases of KFD are typically reported during December–May, the same period during which monkey deaths generally occur. Local public health authorities often undertake precautionary measures on the basis of monkey deaths, including spraying acaricide around areas with monkey carcasses and vaccination of persons within a 5-km radius (*6*,*14*). The importance of animals as sentinels of infectious diseases, environmental hazards, and acts of bioterrorism is well documented (*15*). Because monkey deaths are used as sentinels for KFD, establishing year-round surveillance systems that consistently report KFD-related monkey deaths by date, location, and species is essential to better understand the epidemiology of the disease and design appropriate public health measures.

One limitation of our review is the inconsistency and gaps in the availability of reported monkey deaths caused by KFD. Few studies report monkey mortality data or provide specific monkey deaths by location, year, and species, so assessing whether mortality rates have changed over time is difficult. Another limitation is the incomplete data on testing and diagnoses of monkey carcasses for KFDV because of challenges such as distance to the testing site and delays in discovery. 

Further research is needed to develop serosurveys specific to KFDV among monkeys, determine species-specific vulnerability to KFDV, and assess whether KFDV can spread through routes other than tick transmission. Testing capacity in KFD-endemic states should be strengthened to conduct timely monkey necropsies, providing more information on the prevalence of KFD in these sentinel animals, to elucidate the epidemiology of KFDV and protect monkey and human health.

Appendix Information related to Kyasanur Forest disease and deaths among nonhuman primates.
